# Prediction of Chinese Reading Fluency by Verbal and Non-verbal Visual Attention Span Measures

**DOI:** 10.3389/fpsyg.2019.03049

**Published:** 2020-02-04

**Authors:** Kevin Shing-Chi Chan, Pui-Sze Yeung

**Affiliations:** Faculty of Education, The University of Hong Kong, Pokfulam, Hong Kong

**Keywords:** Chinese reading fluency, global report, visual attention span, visual 1-back, word reading fluency

## Abstract

This study explored (1) the relationship between verbal and non-verbal visual attention span measures and (2) the relationship between visual attention span and reading fluency in traditional Chinese, among 101 university students in Hong Kong. The participants’ visual attention span was assessed using verbal measures (i.e., a global report task and a partial report task) and non-verbal measures (i.e., visual 1-back task with Chinese characters and visual 1-back task with symbols). The results of the confirmatory factor analysis indicated that the single latent factor model, composed of the global report task, the partial report task, the visual 1-back task with Chinese characters, and the visual 1-back task with symbols, was a good fit for the data. The results of the regression analysis showed that the global report task significantly predicted traditional Chinese reading fluency. Structural equation modeling revealed a significant predictive relationship between the single latent factor composed of verbal and non-verbal visual attention span measures and traditional Chinese reading fluency. Overall, the results indicate that visual attention span contributes to reading fluency in traditional Chinese.

## Introduction

Visual attention span has attracted growing research interest in recent years. Studies have shown that visual attention span is a significant predictor of reading ability and difficulty (e.g., Bosse and Valdois, 2003, 2009; Bosse et al., 2007). Visual attention span refers to the number of elements that can be processed in parallel (Bosse and Valdois, 2003; Bosse et al., 2007). Such parallel processing occurs without actual eye movement and with only a brief view of visual elements (Prado et al., 2007; Ziegler et al., 2010; Banfi et al., 2018). According to the multiple-trace memory model of reading (Ans et al., 1998), reading relies on two types of visual word processing procedures: a global procedure and an analytical procedure. The major difference between the two procedures is the kind of visual attention processing involved. The global procedure allows a whole word string to be processed in parallel and makes use of “knowledge about entire words” in reading. The analytical procedure involves the activation and sequential processing of sub-word segments (Ans et al., 1998, p. 678).

A number of studies have shown that visual attention span is independent of phonological awareness and predicts reading over and above phonological skills (Bosse and Valdois, 2003, 2009; Bosse et al., 2007; Peyrin et al., 2011, 2012; Lobier et al., 2012a; Chen et al., 2019). Non-significant correlations have been found between visual attention span and phonological awareness (Bosse and Valdois, 2003; Bosse et al., 2007; Chen et al., 2019). Evidence from factor analysis suggests that phonological awareness and visual attention span belong to two different cognitive constructs (Bosse and Valdois, 2009). Neurological studies have shown that during a visual attention span task, adults with visual attention span deficit demonstrate decreased activation of the parietal lobule (Peyrin et al., 2012), in particular the left superior parietal lobule (Peyrin et al., 2011). In contrast, during phonological tasks, typical adults and adults with visual attention span deficit have shown similar activation of the left inferior frontal gyrus (Peyrin et al., 2012).

Numerous studies have shown that visual attention span is significantly correlated with reading ability in alphabetic languages among children, such as in French, English, Portuguese, Spanish, and Dutch (Bosse et al., 2014; Germano et al., 2014; van den Boer et al., 2015; Chen et al., 2016; Antzaka et al., 2018), and adults, such as in French and Arabic (Awadh et al., 2016; Antzaka et al., 2017). After age, IQ, and phonological awareness are controlled for, visual attention span serves as a unique predictor of the French and English reading accuracy (Bosse et al., 2007; Bosse and Valdois, 2009), French reading fluency (Bosse and Valdois, 2009), and Dutch reading fluency (van den Boer et al., 2013, 2015) of junior-grade children. Not only does visual attention span predict reading ability among typical adult readers, but it also significantly predicts English reading comprehension among adults with dyslexia (Chen et al., 2016).

Less is known about the relationships between visual attention span and reading ability in non-alphabetic writing systems. Only a small number of studies have been conducted on the relationship between visual attention span and reading ability in logographic scripts, such as Kanji in Japanese (Uno and Tsutamori, 2014) and Chinese (Zhao et al., 2017, 2018; Chen et al., 2019). In general, the findings have been mixed. Some studies have shown significant relationships between visual attention span and Chinese reading ability among junior-grade children (Zhao et al., 2018; Chen et al., 2019). However, one study of typical Chinese adults showed a significant relationship between visual attention span and silent sentence reading, but not between visual attention span and oral sentence reading (Zhao et al., 2017).

### Verbal and Non-verbal Visual Attention Span Measures

In the majority of the previous studies on visual attention span, two tasks (i.e., the global report task and the partial report task) that require verbal responses were used to assess the participants’ visual attention spans (Bosse and Valdois, 2003, 2009; Bosse et al., 2007; Germano et al., 2014; van den Boer et al., 2015; Chen et al., 2019). In the global report task, participants are asked to repeat as many letters as possible from a briefly viewed letter array regardless of the order. In the partial report task, participants are asked to repeat a cued letter from a briefly viewed letter array. In general, the performance in verbal visual attention span measures (i.e., the global and partial report tasks) is positively associated with reading ability (Bosse and Valdois, 2003, 2009; van den Boer et al., 2015; Chen et al., 2016, 2019; Antzaka et al., 2017). The performance in the global report task was found to be significantly correlated to reading fluency (Bosse et al., 2007; Bosse and Valdois, 2009), rapid automatized naming (van den Boer et al., 2013, 2015) and reading comprehension (Chen et al., 2016), and a significant predictor of reading fluency and reading comprehension (Bosse et al., 2007; Bosse and Valdois, 2009; van den Boer et al., 2013, 2015; Chen et al., 2016). Performance in the partial report task was also significantly correlated to reading fluency (Awadh et al., 2016).

Some researchers have claimed that verbal visual attention span measures (i.e., the global and partial report tasks) rely on verbal responses to linguistic stimuli; therefore, the performance in both tasks may be confounded by visual-to-verbal mapping abilities (Ziegler et al., 2010; Zhao et al., 2017, 2018; Banfi et al., 2018). As a result, non-verbal visual attention span tasks, which minimize the influence of visual-to-verbal mapping abilities in visual attention span measures, have been developed as “pure” measures of visual attention span (Pammer et al., 2004; Ziegler et al., 2010; Lobier et al., 2012b; Collis et al., 2013; Onochie-Quintanilla et al., 2017; Yeari et al., 2017; Zhao et al., 2017, 2018; Banfi et al., 2018; Lallier et al., 2018). In non-verbal visual attention span tasks, participants are first briefly presented with an array of linguistic or non-linguistic stimuli. Then, the participants are required to respond to a target stimulus by pressing the corresponding key on a computer keyboard. Compared to the number of studies using verbal visual attention span measures, fewer studies have examined the relationships between non-verbal visual attention span and reading ability—their findings have also been less consistent (Pammer et al., 2004; Ziegler et al., 2010; Lobier et al., 2012b; Onochie-Quintanilla et al., 2017; Yeari et al., 2017; Banfi et al., 2018; Lallier et al., 2018; Zhao et al., 2018).

Studies have been conducted to compare the performance of non-verbal visual attention span tasks between readers with dyslexia and typical readers in different writing systems. A significant between-group difference has been found among children in simplified Chinese (Zhao et al., 2018) and in English (Pammer et al., 2004), but not in German (Banfi et al., 2018). The findings among French children have been mixed (Ziegler et al., 2010; Lobier et al., 2012b). No significant between-group difference was found among adults with and without dyslexia in Hebrew (Yeari et al., 2017) or in English (Collis et al., 2013). Other studies have examined the correlation between non-verbal visual attention span and reading ability. Significant correlations between the performance of non-verbal visual attention span tasks and reading ability have been found among children in English (Pammer et al., 2004), in French (Lobier et al., 2012b), and in Spanish (Onochie-Quintanilla et al., 2017), but not in Arabic (Lallier et al., 2018). Due to the limited number of published studies using non-verbal attention span tasks, little or no replication of the results in each writing system has been conducted. Furthermore, very few studies have been conducted on Chinese readers (Zhao et al., 2017).

### Chinese Writing Systems

Chinese orthography is logographic (Chung and Ho, 2010). Chinese characters are square shaped, and almost all are the same size. Chinese orthography does not rely on grapheme–phoneme conversion rules, which means that the phoneme of a Chinese character cannot be directly retrieved from its grapheme (Perfetti and Tan, 1998). Although the sound of a Chinese character can be predicted from its phonetic radical, the accuracy of the correct pronunciation of an ideophonetic compound character based on its phonetic radical is less than 30% when tone is considered (Zhou, 1980; Fan, 1986; Shu et al., 2003; Chung and Leung, 2008).

The shape of Chinese characters and the lack of reliable grapheme–phoneme conversion rules mark the difference between Chinese and alphabetic languages, such as English and French. In general, processing Chinese characters requires more visual resources than alphabetic languages (McBride, 2016). As Chinese characters have two-dimensional and visually complex structures, a fine-grained detection and identification process is needed for character recognition (Liu, 2014). In addition, no clear word boundary exists for multicharacter Chinese words (Chen et al., 2019). Therefore, reading Chinese characters may require more visual attentional recourses.

Chinese-speaking populations have two Chinese writing systems (Liu and Hsiao, 2012): traditional Chinese, which is commonly used in Hong Kong and Taiwan, and simplified Chinese, which is commonly used in mainland China. Simplified Chinese characters are generally less visually complex than traditional Chinese characters, but more visually similar to other simplified Chinese characters (Chen, 1999). Children in Hong Kong learn to read traditional Chinese with a whole-character approach, associating the word and the sound of Chinese characters, without the assistance of a phonetic system. However, children in mainland China learn to read simplified Chinese with an alphabetic coding system, called the pinyin system (McBride-Chang et al., 2005).

Due to their different learning experiences, different cognitive processes have been found between readers of traditional and simplified Chinese (McBride-Chang et al., 2005; Liu and Hsiao, 2012). A study of adults from China and Hong Kong found that readers of simplified Chinese use a more analytical process than readers of traditional Chinese for reading simplified Chinese characters and characters shared in simplified and traditional Chinese scripts (Liu and Hsiao, 2012). As a result of the lower visual complexity of simplified Chinese than that of traditional Chinese characters (Chen, 1999), expert readers of simplified Chinese may adopt a more analytic visual process for word recognition.

As mentioned, processing Chinese generally requires more visual resources (Liu, 2014; McBride, 2016). As there is no clear boundary between multiple Chinese characters, expert readers of Chinese may have to process multiple Chinese characters simultaneously for word recognition and further linguistic processing. As a result, it is comparable to the mechanism of visual attention span in alphabetic languages, except the level of stimulus involves characters rather than letters.

### Visual Attention Span and Reading in Chinese

By facilitating readers’ ability to distinguishing multiple Chinese characters simultaneously (Chen et al., 2019), visual attention span may help enhance reading speed. Indirect evidence from studies showing that the performance of visual attention span tasks among students with dyslexia is significantly worse than that of typical students in both simplified and traditional Chinese supports this hypothesis (Zhao et al., 2018; Chen et al., 2019). However, only two studies on the contribution of visual attention span to reading ability in Chinese have been published. The performance of visual attention span has been shown to be a contributing factor to oral reading ability in traditional Chinese characters among a group of children in Hong Kong (Chen et al., 2019), but visual attention span has been shown to not significantly predict oral reading in simplified Chinese among adults in Beijing (Zhao et al., 2017). This study aims to enrich the research area concerning the relationship between visual attention span and Chinese reading ability, especially in traditional Chinese.

### Research Goals

Evidence has shown that performance in the verbal and non-verbal visual attention span measures is significantly correlated (Lobier et al., 2012b) and that verbal visual attention span tasks mainly tap into visual abilities, not verbal abilities. A limitation in the field is that most, if not all, studies exploring the relationship between visual attention span and reading ability have used either verbal or non-verbal visual attention span measures. The paucity of studies using both verbal and non-verbal measures makes it difficult, if not impossible, to compare the predictive abilities between verbal and non-verbal visual attention span measures on reading. This study aims to fill this gap in the literature.

This study aims to address two research questions. First, what are the relationships between the different measures of visual attention span? Second, does visual attention span predict reading ability in traditional Chinese script? Regarding the first research question, various methods have been used to measure visual attention span. Studies have reported significant correlations between the global and partial report tasks (Bosse and Valdois, 2009) and between the visual 1-back tasks with linguistic stimuli and with symbols (Banfi et al., 2018). However, only one study has explored the relationship between verbal and non-verbal measures of visual attention span (Lobier et al., 2012b). This study aims to enrich the research by examining the relationship between performance in verbal report tasks (i.e., the global and partial report tasks) and non-verbal report tasks (i.e., visual 1-back tasks with verbal stimuli and with symbols) of visual attention span. It is hypothesized that both types of measures are valid measures of visual attention span despite the different nature of the responses. Significant intercorrelations are expected between all visual attention span measures. This study also hypothesizes that the global report task, the partial report task, the visual 1-back task with linguistic stimuli, and the visual 1-back task with symbols regress on one single latent factor measuring visual attention span.

Concerning the second research question, two studies have examined the relationships between visual attention span and reading ability in simplified Chinese and have found significant correlations between visual attention span and reading fluency (Zhao et al., 2017, 2018). However, such findings have not been established for traditional Chinese. As simplified Chinese and traditional Chinese are both logographic writing systems, this study hypothesizes that the correlation between visual attention span and traditional Chinese reading fluency is significant. Furthermore, all visual attention span measures (i.e., the global report task, partial report task, visual 1-back task with Chinese characters, and visual 1-back task with symbols) are expected to be intercorrelated. It is also hypothesized that verbal and non-verbal measures of visual attention span (i.e., the global report task, partial report task, visual 1-back task with Chinese characters, and visual 1-back task with symbols) significantly predict traditional Chinese reading fluency.

## Materials and Methods

### Participants

The participants included 101 university students (66 females; age, *M* = 23.73 years, *SD* = 4.38 years) in Hong Kong. All of the participants were native Cantonese speakers who were fluent speakers of English with normal or corrected-to-normal vision. None of the participants reported reading difficulties.

### Materials and Procedure

The participants were administrated two verbal visual attention span tasks (i.e., a global report and a partial report), two non-verbal visual attention span tasks (i.e., visual 1-back with Chinese characters and visual 1-back with symbols), and two Chinese reading fluency tasks (i.e., word reading fluency and text reading fluency). The visual attention span measures and administrating procedure were developed based on previous studies (Bosse and Valdois, 2009; Chen et al., 2016; Antzaka et al., 2017; Banfi et al., 2018). As the participants of this study were adults, they received six stimuli in the visual attention span measures (Chen et al., 2016; Antzaka et al., 2017) rather than the five typically delivered to junior-grade participants (Bosse and Valdois, 2009; van den Boer et al., 2015).

The experiment took approximately 40 min to complete and was conducted inside a quiet room in the library at The University of Hong Kong. The experiment was conducted with a fixed measure order for all of the participants. The global report task and the partial report task were administrated followed by the Chinese reading fluency tasks and the visual 1-back tasks. For the visual attention span tasks, a visual experiment software package, OpenSesame 3.1 with the xpyriment back-end setting (Mathôt et al., 2012), was administrated on a 12-in. Apple MacBook with a display resolution set to 1,024 × 800. The MacBook was taken off-line, and its other applications were turned off to ensure stable stimulus presentation. The participants sat approximately 50 cm away from the MacBook with a viewing angle of approximately 4.70° for the visual attention span measure trials. For the global and partial report tasks, the participants pressed the spacebar on the keyboard to proceed to the next trial. For the visual 1-back tasks, the next trial began immediately after the participants pressed the *z* or *m* key.

This study was approved by the Ethics Committee of the University of Hong Kong (reference: EA1801030). Informed written consent was obtained from each participant, who each received HK$50 cash for participating in the experiment.

#### Global Report Task

The six-character strings used in the global report task consisted of different combinations of 10 high-frequency traditional Chinese characters, each composed of eight strokes (i.e., 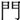, 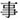, 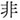, 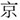, 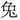, 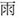, 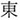, 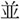, 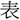, and 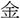) without repetition within a string. The six-character strings were also not repeated within this task. All of the characters were developed with a fixed-width font, Song Ti with a font size of 22. The distance between the characters was set to 0.5 cm to minimize lateral masking. Each character appeared in the same position twice. A total of 20 trials were conducted for the global report task preceded by two practices.

**FIGURE 1:**
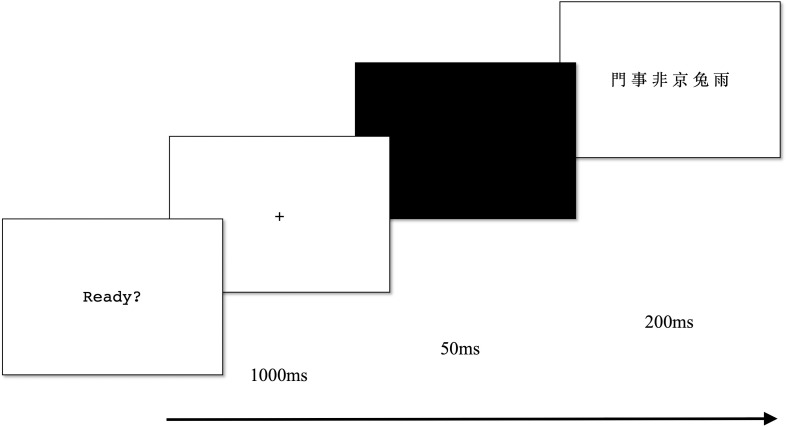
Global report task procedure.

Each trial began with a central fixation (1,000 ms) followed by a black screen (50 ms) and a centrally displayed character string (200 ms). The character string was presented in black on a white background. Following the character-string presentation, the participants were instructed to verbally report as many characters as possible regardless of order. Figure 1 illustrates the procedure of the global report task. The experimenter marked the reported characters on an answer sheet and proceeded to the next trial by pressing a button without giving feedback. The individual score was the sum of the correct reported characters (max = 120). This task was found to be highly reliable (α = 0.83).

#### Partial Report Task

The six-character strings used in the partial report task consisted of different combinations of six traditional Chinese characters each composed of eight strokes (i.e., 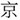, 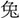, 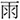, 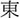, 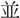, and 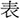) without repetition within a string. The six-character strings were not repeated within this task and were not repeated from the global report task. All of the characters were developed with a fixed-width font, Song Ti with a font size of 22. The distance between the characters was set to 0.5 cm to minimize lateral masking. Each character appeared six times in the same position and served as the target character once at each position. A total of 36 trials were conducted for the partial report task preceded by two practices.

**FIGURE 2:**
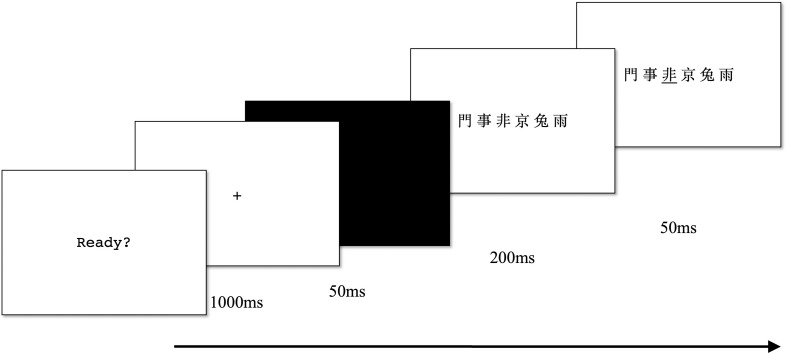
Partial report task procedure.

Each trial began with a central fixation (1,000 ms) followed by a black screen (50 ms) and a centrally displayed character string (200 ms). This was followed by the presentation of a black underline (50 ms) cueing the target character. Figure 2 illustrates the procedure of the partial report task. The procedure was slightly modified from previous partial report tasks in that a cue appears after the stimuli. The participants were instructed to only report the target character. The experimenter marked the reported character on an answer sheet and proceeded to the next trial by pressing a button without giving feedback. The individual score was the sum of the correct reported target characters (max = 36). This task was also found to be highly reliable (α = 0.79).

#### Visual 1-Back Tasks

The participants were administrated two visual 1-back tasks (i.e., Chinese characters and symbols) to measure their non-verbal visual attention span.

The six-character strings in the visual 1-back task with Chinese characters were different combinations of eight high-frequency traditional Chinese characters (i.e., 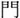, 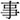, 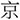, 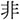, 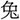, 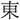, 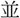, and 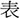) without repetition within a string. Four characters also served as the target characters (i.e., 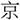, 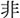, 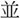, and 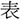). The six-character strings were not repeated within this task and were not repeated from the global report task or the partial report task. All of the characters were developed with a fixed-width font, Song Ti, with a font size of 22. The distance between the characters was set to 0.5 cm to minimize lateral masking. Each character appeared six times in the same position. A total of 48 trials were conducted for this task preceded by four practices.

The six-symbol strings, in the visual 1-back task with symbols, consisted of different combinations of eight unfamiliar symbols (i.e., 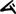, 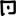, 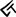, 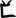, 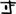, 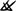, 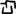, and 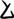) without repetition within a string, and four symbols also served as the target symbols (i.e., 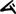, 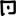, 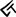, and 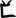). The six-symbol strings were not repeated within this task. The distance between the stimuli was set to 0.5 cm to minimize lateral masking. Each symbol appeared six times at the same position. A total of 48 trials were conducted for this task preceded by four practices.

**FIGURE 3:**
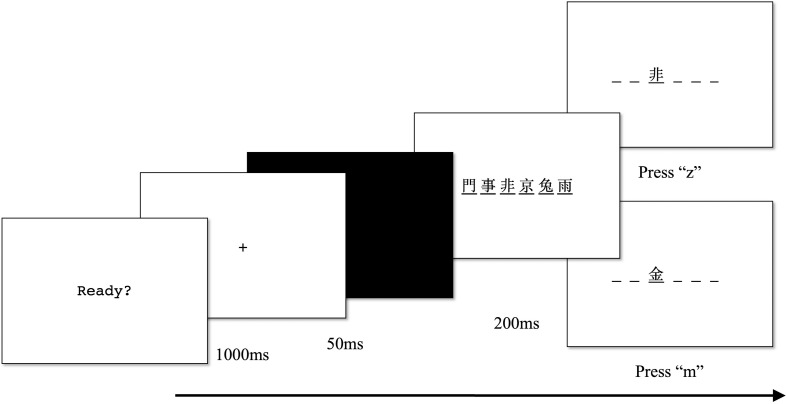
Visual 1-back task procedure.

Each trial began with a central fixation (1,000 ms) followed by a black screen (50 ms) and a centrally displayed six-stimulus string with a black underline below each stimulus (200 ms). This was followed by the presentation of a target stimulus. For half of the trials, the target stimulus was shown at the same position as in the stimulus string. For the other half of the trials, the target stimulus was not shown in the stimulus string and presented in a random position. The participants were instructed to state whether the target stimulus was shown at the same position as in the stimulus string by pressing *z* (same position) or *m* (not the same position) on the keyboard. Figure 3 illustrates the procedure for the visual 1-back tasks. The individual sensitivity index (*d’*) was calculated based on the hit rates and the false alarm rates of each participant [*d’* = *z* (hit rate) − *z* (false alarm rate)]. Both visual 1-back tasks were found to be highly reliable (Chinese characters, α = 0.83; symbols, α = 0.81).

#### Chinese Reading Fluency

A word list consisting of 120 two-character high-frequency traditional Chinese words was developed for the word reading fluency task. The word list was printed on two side-by-side white A4 papers in black KaiTi font with a font size of 22. A passage consisting of 420 high-frequency traditional Chinese words was constructed for the text reading fluency task. The passage was printed on a white A4 paper in black KaiTi font with a font size of 16.

For both reading fluency tasks, the participants were instructed to read aloud as many of the words as accurately as possible within 45 s. The experimenter timed 45 s and marked mispronounced Chinese words. One mark was given for each correctly pronounced Chinese word within 45 s. The total score was the sum of Chinese words correctly pronounced within 45 s (word fluency, max = 120; text fluency, max = 420). All but eight of the participants finished the word list of word reading fluency within the required time. Their scores were calculated based on the proportion of 45 s [score = (number of correctly read words/finishing time) ^∗^ 45]. None of the participants finished the passage of text reading fluency within 45 s.

As the reading fluency tasks were designed as time-limited tasks, the individual scores were expected to relate to speed. Gulliksen’s formula (*H*^2^ = variance of attempted questions/total variance) was used to estimate the attribution of speed for this task, with *H*^2^ values larger than 0.2 considered to be attributed to speed (Gulliksen, 1950; Anastasi and Drake, 1954). The *H*^2^ of this task indicated that the task was highly attributed to speed (word fluency, *H*^2^ = 0.88; text fluency, *H*^2^ = 1), consistent with the task’s design. Due to the high attribution of speed, a stable internal consistency could not be generated (Gulliksen, 1950; Anastasi and Drake, 1954). According to Gulliksen (1950), a lower bound of internal consistency could be estimated by the mean and variance [1 - (mean/variance)] of a time-limited task. The lower-bound reliability of this task was calculated (word fluency, *r*_lower_ = 0.66; text fluency, *r*_lower_ = 0.90).

## Analyses

Descriptive statistics, correlations, exploratory factor analysis, and regression analysis were conducted using a data analysis software package, jamovi 1.1.5.0 (The jamovi project, 2019). Structural equation modeling was conducted using another data analysis software package, R 3.6.0 (R Core Team, 2019) with the lavaan 0.6–5 structural equation modeling package (Rosseel, 2012).

An exploratory factor analysis involving maximum likelihood estimation with varimax rotation was conducted to examine the factor structure of age and visual attention span. Chi-square (χ^2^) was used to determine the model fit of the factor structure. A significant χ^2^ (*p* < 0.05) indicates that there is no significant difference between the null model and the proposed model, demonstrating a poor fit to the data (Ropovik, 2015). Confirmatory factor analyses were conducted to examine the factor structure of various visual attention span measures. χ^2^ and absolute fit indices, including the comparative fit index (*CFI*), the standardized root mean square residual (*SRMR*), and the root mean square error of approximation (*RMSEA*), were reported to indicate the fit to the data. A *CFI* larger than 0.95 and an *SRMR* below 0.08 indicate a good fit to the data (Hu and Bentler, 1999). An *RMSEA* below 0.05 also indicates a good fit to the data (Browne and Cudeck, 1993).

The comparison of Bayesian information criterion (*BIC*) values and a χ^2^ test of model difference was used to determine a better model for the data. A significant χ^2^ test of model difference indicates that the model with fewer degrees of freedom (*df*) fits the data better (Kline, 2011). A lower *BIC* value shows that there is less loss of information in a latent model. A negative two-unit *BIC* value has considerably less loss of information, which indicates a potentially better fit to the data (Sakamoto et al., 1988; Kershaw and Schatschneider, 2012).

## Results

Table 1 shows the descriptive statistics of the measures. No values were missing, and no outliner existed for any of the measures. For the global report task, the partial report task, and the two Chinese reading fluency tasks, a zero score means that a participant did not provide any correct responses on those measures. However, for the visual 1-back tasks, a zero *d’* value means that a participant made equal hit rates and false alarm rates in that task. It does not necessarily mean that the participants did not make any correct response. A negative value of *d’* means that a participant made more false alarms than hits, whereas a positive value means that a participant made more hits than false alarms.

**TABLE 1:** Descriptive statistics.

	**Global**	**Partial**	**V1B-C**	**V1B-S**	**CWR**	**CTR**
*N*	101	101	101	101	101	101
*M*	73.57	23.78	0.86	0.91	96.06	232.50
*SD*	10.67	5.59	0.50	0.56	16.77	47.38
*Min*	51	7	−0.67	−0.43	57	100
*Max*	96	34	2.04	2.53	137	348
*Reliability*	0.83*^*a*^*	0.79*^*a*^*	0.83*^*a*^*	0.81*^*a*^*	0.66*^*b*^*	0.90*^*b*^*

**^*a*^*Cronbach’s alpha. *^*b*^*Time-limited task, lower bound of reliability. Global = Global report task; Partial = Partial report task; V1B-C = Visual 1-back task with Chinese characters; V1B-S = Visual 1-back task with symbols; CWR = Chinese word reading fluency; CTR = Chinese text reading fluency.*

### Relationship Between Various Visual Attention Span Measures

Table 2 shows the correlations between all of the study measures. The partial correlations between all of the visual attention span measures, with age controlled, were significant (0.21 < *r*s < 0.49). The visual attention span measures with linguistic stimuli (i.e., the global report task, partial report task, and visual 1-back task with Chinese characters) were moderately correlated (0.31 < *r*s < 0.49), whereas the visual attention span measure with non-linguistic stimuli was weakly but significantly correlated with the other visual attention span measures (0.21 < *r*s < 0.25).

**TABLE 2:** Partial correlations with age controlled.

	**Global**	**Partial**	**V1B-C**	**V1B-S**	**CWR**
Partial	0.49***				
V1B-C	0.30**	0.31**			
V1B-S	0.22*	0.21*	0.25*		
CWR	0.24*	0.22*	0.16	0.15	
CTR	0.24*	0.13	0.15	0.15	0.80***

*p < 0.05, **p < 0.01, ***p < 0.001. Global = Global report task; Partial = Partial report task; V1B-C = Visual 1-back task with Chinese characters; V1B-S = Visual 1-back task with symbols; CWR = Chinese word reading fluency; CTR = Chinese text reading fluency.

Exploratory factor analysis involving maximum likelihood estimation with varimax rotation was used to explore the factor structure among age and all visual attention span measures (i.e., the global report task, partial report task, visual 1-back task with Chinese characters, and visual 1-back task with symbols). The results showed a good fit to the data [χ^2^(1, *n* = 101) = 1.49, *p* > 0.05]. Two factors were extracted based on their eigenvalues. Table 3 shows the factor loadings among age and all visual attention span measures. Factor 1 consisted of all visual attention span measures (i.e., the global report task, partial report task, visual 1-back task with Chinese characters, and visual 1-back task with symbols), whereas factor 2 consisted solely of age.

**TABLE 3:** Exploratory factor analysis involving maximum likelihood with varimax rotation among age and visual attention span.

**Variables**	**Factor 1**	**Factor 2**
Age	−0.10	0.99
Global report	0.69	0.03
Partial report	0.69	–0.16
Visual 1-back Chinese	0.46	–0.05
Visual 1-back symbols	0.35	–0.18

**FIGURE 4:**
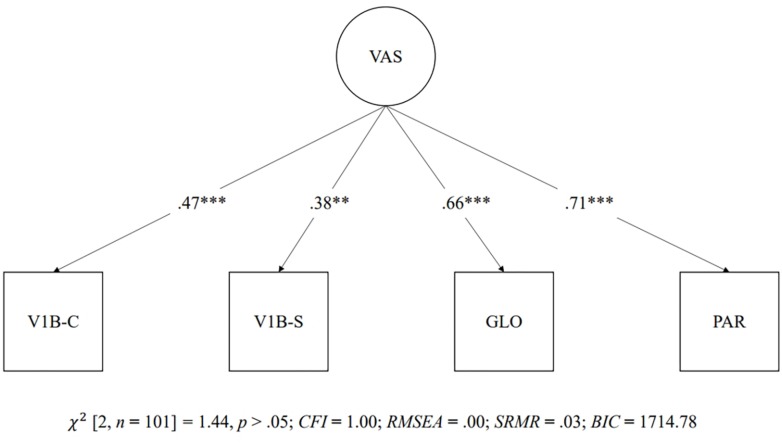
One latent factor model of visual attention span measures. ^∗∗∗^*p* < 0.001. GLO = Global report task; PAR = Partial report task; V1B-C = Visual 1-back task with Chinese characters; V1B-S = Visual 1-back task with symbols.

**FIGURE 5:**
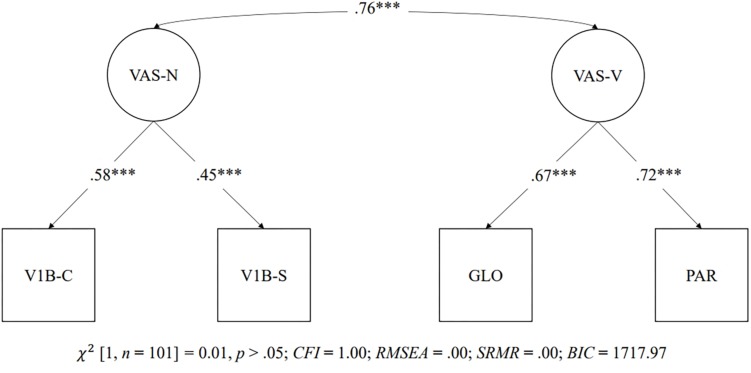
Two latent factor models of visual attention span measures. ^∗∗∗^*p* < 0.001. GLO = Global report task; PAR = Partial report task; V1B-C = Visual 1-back task with Chinese characters; V1B-S = Visual 1-back task with symbols.

Confirmatory factor analysis was used to test three models conceptualizing the relationships between the visual attention span measures. The first latent model showed a single latent factor made up of all of the visual attention span measures (i.e., the global report task, partial report task, visual 1-back task with Chinese characters, and visual 1-back task with symbols). Figure 4 shows the path diagram of the single latent factor model, which showed a good fit to the data [χ^2^(2, *n* = 101) = 1.44, *p* > 0.05; *CFI* = 1.00; *RMSEA* = 0.00; *SRMR* = 0.03; *BIC* = 1,714.78]. The second latent model consisted of two intercorrelated latent factors. One factor was made up of verbal visual attention span measures (i.e., the global and partial report tasks), and the other factor was made up of non-verbal visual attention span measures (i.e., visual 1-back with Chinese characters and visual 1-back with symbols). Two latent variables were significantly correlated (*r* = 0.76, *p* < 0.001). Figure 5 shows the path diagram of the two intercorrelated latent factor model, which showed a good fit to the data [χ^2^(1, *n* = 101) = 0.01, *p* > 0.05; *CFI* = 1.00; *RMSEA* = 0.00; *SRMR* = 0.00; *BIC* = 1,717.97]. The third latent model consisted of one latent factor made up of the three visual attention span measures with Chinese characters (i.e., the global report task, partial report task, and visual 1-back task with Chinese characters). The significant χ^2^ in model 3 indicated a poor fit to the data [χ^2^(0, *n* = 101) = 0.00, *p* < 0.05].

As models 1 and 2 showed a good fit index, a χ^2^ test of model difference and the comparison of *BIC* values were used for model selection between models 1 and 2. The χ^2^ test of model difference indicated that there was no significant difference between models 1 and 2 [χ^2^(1) = 1.43, *p* > 0.05]. The *BIC* values for models 1 (i.e., a single factor made up of all visual attention span measures) and 2 (i.e., one factor made up of verbal visual attention span measures and one factor made up of non-verbal visual attention span measures) were 1,714.78 and 1,717.97, respectively. Therefore, despite the non-significant χ^2^ difference between models 1 and 2, the negative three-unit *BIC* value in the first model (i.e., a single factor made up of all visual attention span measures) indicated less loss of information in model 1 and that the visual attention span measures fit better in a single latent factor model.

### Prediction of Chinese Word Reading Fluency

Table 2 shows the results for the partial correlation between Chinese reading fluency and visual attention span with age controlled. Significant correlations were found between the global report and Chinese reading fluency (*r* = 0.24, *p* < 0.05). Chinese word reading fluency was also significantly correlated with the partial report (*r* = 0.22, *p* < 0.05). However, the visual 1-back tasks were not significantly correlated with either word reading fluency or text reading fluency.

Regression analysis was conducted to test the predictive relationship between visual attention span and Chinese reading fluency. Age was entered in the first step, and visual attention span measures were entered in the second step separately. Table 4 shows the predictive relationship between word reading fluency and visual attention span. Table 5 shows the predictive relationship between text reading fluency and visual attention span. The global report explained 6% of the variance in Chinese word reading fluency (*p* < 0.05) and significantly predicted performance in Chinese word reading fluency (β = 0.24, *t* = 2.41, *p* < 0.05). The global report also explained 6% of the variance in Chinese text reading fluency (*p* < 0.05) and significantly predicted performance in the Chinese text reading fluency task (β = 0.24, *t* = 2.47, *p* < 0.05). The partial report explained 5% of the variance (*p* < 0.05) in Chinese word reading fluency and significantly predicted performance in the Chinese word reading fluency task (β = 0.22, *t* = 2.18, *p* < 0.05). In contrast, the change in *R*^2^ for non-verbal visual attention span (i.e., visual 1-back with Chinese characters and visual 1-back with symbols) was not significant. When the global report and partial report together entered in step 2, they explained 7% of the variance in Chinese word reading fluency (*p* < 0.05). However, neither the global report nor the partial report significantly predicted the performance of the Chinese word reading fluency task in this model.

**TABLE 4:** Regression analysis on Chinese word reading fluency.

		***R*^2^ change**	***F* change**	**β**	***T***
1	Age	0.00	0.06^*NS*^	–0.03	−0.25^*NS*^
2	Global report	0.06	5.82*	0.24	2.41*
2	Partial report	0.05	4.75*	0.22	2.18*
2	Visual 1-back Chinese	0.03	2.57^*NS*^	0.16	1.60^*NS*^
2	Visual 1-back symbols	0.02	2.26^*NS*^	0.15	1.50^*NS*^
2	Global report	0.07	3.60*	0.17	1.54^*NS*^
	Partial report			0.13	1.16^*NS*^

*p < 0.05; *NS* = non-significant.

**TABLE 5:** Regression analysis on Chinese text reading fluency.

		***R*^2^ change**	***F* change**	**β**	***T***
1	Age	0.00	0.08^*NS*^	–0.02	−0.28^*NS*^
2	Global report	0.06	6.09*	0.24	2.47*
2	Partial report	0.02	1.68^*NS*^	0.13	1.30^*NS*^
2	Visual 1-back Chinese	0.02	2.10^*NS*^	0.15	1.45^*NS*^
2	Visual 1-back symbols	0.02	2.24^*NS*^	0.15	1.50^*NS*^

*p < 0.05; **NS** = non-significant.

**FIGURE 6:**
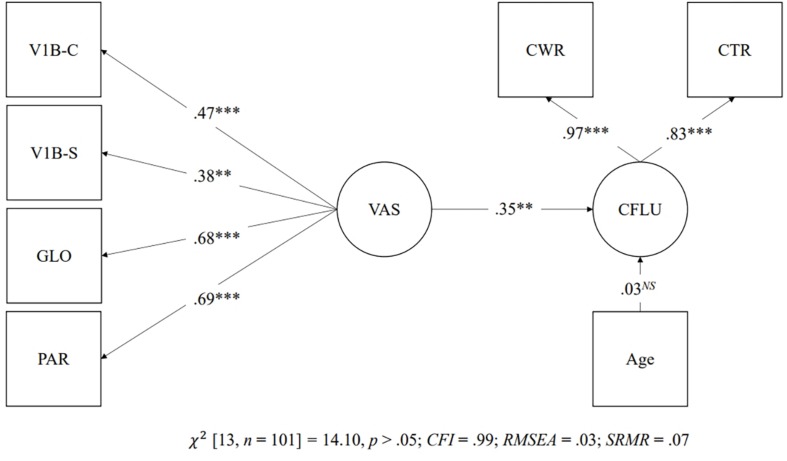
Prediction of Chinese reading fluency by the one latent factor model. ^∗∗^*p* < 0.01, ^∗∗∗^*p* < 0.001, *NS* = non-significant. GLO = Global report task; PAR = Partial report task; V1B-C = Visual 1-back task with Chinese characters; V1B-S = Visual 1-back task with symbols; CWR = Chinese word reading fluency; CTR = Chinese text reading fluency.

**FIGURE 7:**
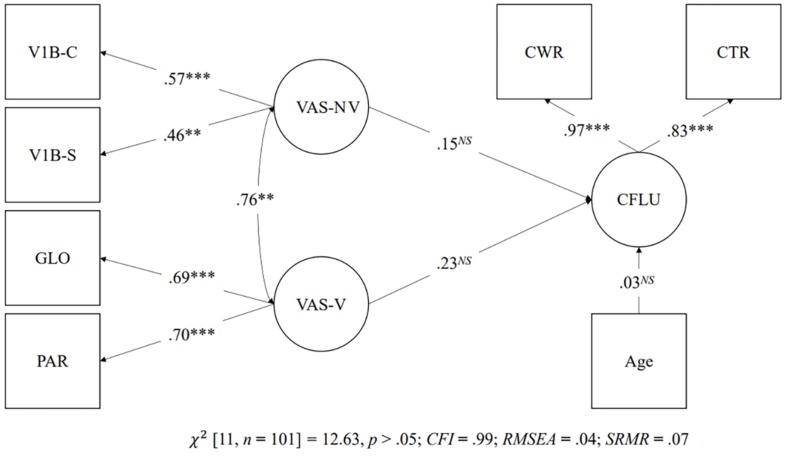
Prediction of Chinese reading fluency by the two latent factor models. ^∗∗^*p* < 0.01, ^∗∗∗^*p* < 0.001, *NS* = non-significant. GLO = Global report task; PAR = Partial report task; V1B-C = Visual 1-back task with Chinese characters; V1B-S = Visual 1-back task with symbols; CWR = Chinese word reading fluency; CTR = Chinese text reading fluency.

Structural equation modeling was used to test the predictive relationship between the latent factor of visual attention span and Chinese reading fluency. As mentioned, visual attention span was examined with two latent models. The first latent model consisted of one latent factor made up of all visual attention span measures, and the second latent model consisted of one latent factor made up of verbal visual attention span measures and one latent factor made up of non-verbal visual attention span measures. The dependent latent variable was made up of the Chinese word reading fluency task and the Chinese text reading fluency task, which entered in the two latent models to examine the predictive relationship between visual attention span and Chinese reading fluency. Both the first latent model [χ^2^(13, *n* = 101) = 14.10, *p* > 0.05; *CFI* = 0.99; *RMSEA* = 0.03; *SRMR* = 0.07] and the second latent model [χ^2^(11, *n* = 101) = 12.63, *p* > 0.05; *CFI* = 0.99; *RMSEA* = 0.04; *SRMR* = 0.07] demonstrated a good fit to the data. Figure 6 shows the predictive relationship of the first latent model. There was a significant predictive relationship between the single latent factor of visual attention span and Chinese reading fluency (β = 0.35, *p* < 0.01). However, as shown in Figure 7, the predictive relationships between verbal visual attention span and Chinese reading fluency word (β = 0.23, *p* = *ns*) and between non-verbal visual attention span and Chinese reading fluency (β = 0.15, *p* = *ns*) were not significant.

## Discussion

### Relationship Between Various Visual Attention Span Measures

This study examines the relationships between different visual attention span measures, including the global report task (Bosse and Valdois, 2003; Bosse et al., 2007), the partial report task (Bosse and Valdois, 2003; Bosse et al., 2007), and the visual 1-back tasks (Zhao et al., 2017, 2018; Banfi et al., 2018). As mentioned, the global report task and the partial report task rely on verbal responses to Chinese characters. The visual 1-back tasks rely on non-verbal responses to both Chinese characters and symbols. This study marks the first attempt to examine the relationship between verbal and non-verbal measures for visual attention span and their role in Chinese reading fluency.

The significant (weak to moderate) correlations found between the verbal and non-verbal measures of visual attention span support the hypothesis that different visual attention span measures are intercorrelated despite the nature of stimuli and the reporting method of the tasks. Furthermore, the exploratory factor analysis demonstrated a two-factor solution for the variables in this study, factor 1 (i.e., the global report task, partial report task, visual 1-back task with Chinese characters, and visual 1-back task with symbols) and factor 2 (i.e., age).

Three latent models were further tested to determine the structure of the visual attention span in Chinese. The single latent factor model supports the hypothesis. It is also consistent with the result of the exploratory factor analysis, showing that verbal and non-verbal visual attention spans belong to the same latent construct despite the reporting method of the task. In a previous study, the global report was significantly correlated with a non-verbal visual task (i.e., non-verbal categorization task; Lobier et al., 2012b). This study further reveals the relationship between verbal visual attention span and non-verbal visual attention span using structural equation modeling, in which both verbal visual attention span and non-verbal visual attention span share amounts of variance and possibly measure the same underlying construct (i.e., visual attention span).

### Prediction of Chinese Reading Fluency

The visual attention span hypothesis (Bosse and Valdois, 2003) posits a relationship between visual attention span and reading ability. Few studies have examined the relationship between visual attention span and Chinese reading ability (Zhao et al., 2017, 2018; Chen et al., 2019). Chen et al. (2019) examined the relationship in traditional Chinese. This study provides more evidence of the role of visual attention span in traditional Chinese reading fluency (i.e., word and text reading fluency).

First, this study shows that the global report is a contributing factor to reading in non-alphabetic languages. Previous studies have mainly examined the contribution of the global report in alphabetic languages (van den Boer et al., 2013, 2014, 2015; Awadh et al., 2016). The results of such studies have consistently shown that the global report is a significant factor in reading among alphabetic languages. Studies in alphabetic languages have confirmed that the number of simultaneous processing elements in a multielement array is significant for reading. This study extends the literature to Chinese and supports the claim that the simultaneous processing of multiple visually complex elements (Chen et al., 2019) is a significant factor of reading fluency in Chinese.

Second, this study extends the literature to adult readers. Previous studies have mainly focused on junior-grade children (Bosse and Valdois, 2003, 2009; van den Boer et al., 2015; Zhao et al., 2018; Chen et al., 2019). Very few studies have been conducted among adults (Awadh et al., 2016; Chen et al., 2016). This study’s findings provide more evidence that the predictive relationship between visual attention span and reading fluency persists among typical adult readers. The explained variance of visual attention span in traditional Chinese reading fluency among adults was similar to the findings among children, specifically 5% in word reading fluency and 4% in text reading fluency (Chen et al., 2019) compared to the 6% in word reading fluency and 6% in text reading fluency in this study. These results indicate that the significance of visual attention span in reading Chinese, particularly in traditional Chinese, is found not only in children but also in adults. Furthermore, correlations of similar magnitude have been found between the global report and reading fluency among children, 0.38 < *r*s < 0.59 (Bosse et al., 2014), and adults, *r* = 0.46 in French (Awadh et al., 2016). The similar pattern across children and adults among Chinese and French suggests that the relationship between visual attention span reading may be universal and is not limited to a particular orthography. This study also sheds light on the relationship between visual attention span and reading fluency in Chinese, suggesting the significant role of visual attention span in logographic languages.

The relationships between the visual 1-back tasks and traditional Chinese reading fluency were also explored. As mentioned, reading simplified Chinese and reading traditional Chinese entail different cognitive processes (McBride-Chang et al., 2005; Liu and Hsiao, 2012). The non-significant correlations found between the visual 1-back tasks (Chinese characters and symbols) and traditional Chinese reading fluency are consistent with the findings in simplified Chinese among adults (Zhao et al., 2017). The findings suggest that Chinese reading ability may be more related to verbal visual attention span. As other visual paradigms have been conducted to measure visual attention span (Lobier et al., 2012b; Yeari et al., 2017), future studies may consider examining the relationship between visual attention span and Chinese reading ability with other visual paradigms. The fact that the predictive abilities of the verbal and non-verbal visual attention span measures for reading fluency were different supports the need to further explore the processes and abilities underlying different measures of visual attention span and their relationships with reading.

The non-significant relationship between non-verbal visual attention span measures and Chinese reading ability among adults revealed in this study and past studies contrasts with the findings from a previous study among children, which identified a group of visual attention span deficit children by non-verbal visual attention span measures in simplified Chinese (Zhao et al., 2018). The differences in the results of studies among adults and children suggest that developmental changes may play a role in the relationships between visual attention span and Chinese reading ability.

### Future Directions and Study Limitations

Future studies could explore the relationship between visual attention span and reading ability in three directions. As processing Chinese characters requires more visual attention resources, the large number of strokes in characters may lead to a “stroke overload” effect (McBride-Chang et al., 2005). Therefore, it would be noteworthy to explore the relationship between visual attention span and the complexity of Chinese characters to determine whether more visually complex characters limit the extent of the visual attention span. Furthermore, the visual processing of Chinese characters between readers of simplified Chinese and traditional Chinese differs (McBride, 2016). Thus, future studies could be conducted to compare the visual attention span of readers of simplified Chinese and of readers of traditional Chinese using characters that exist in both Chinese scripts. A significant difference between the two groups of readers would be evidence of the different visual processing of Chinese characters between simplified and traditional Chinese readers.

A previous study in French and English (Bosse and Valdois, 2009) identified a group of dyslexic children with a visual attention span deficit but not a phonological deficit. Another study found the reading comprehension of a group of adults with dyslexia to be correlated to visual attention span (Chen et al., 2016). Recent studies have found a group of children with visual attention span deficit in simplified Chinese (Zhao et al., 2018) and traditional Chinese (Chen et al., 2019). However, relevant research has yet to be conducted among Chinese adults with dyslexia. Further research could attempt to explore the potential role of visual attention span deficit in Chinese adults with dyslexia.

A number of studies have also shown the significant correlation between the performance of visual attention span and reading ability (Bosse and Valdois, 2009; Lobier et al., 2012b; Chen et al., 2016, 2019). Further experimental research could aim to improve the size of the visual attention span of readers through training to examine the causal relationship between visual attention span and reading.

One limitation of this study was that it did not examine the performance of other linguistic measures, such as rapid naming, reading accuracy, and reading comprehension. Future research could consider examining the relationship between visual attention span and other linguistic measures among Chinese adults. Another limitation concerned participant fatigue during the experiment. The experiment’s administration procedure was fixed for all of the participants. That is, the verbal visual attention span measures were conducted before the non-verbal visual attention span measures. Therefore, during the non-verbal tasks, the participants may have already felt fatigued, and their performance may have been underestimated. Future studies could consider using a block design procedure whereby half of the participants complete the verbal visual attention span tasks before the non-verbal visual attention span tasks, while the other half complete the non-verbal visual attention span tasks before the verbal visual attention span tasks to counterbalance the underestimated performance influenced by fatigue.

Despite these limitations, this study contributes to the literature by examining the relationship between various measures of visual attention span, including both verbal and non-verbal measures of visual attention span. The verbal and non-verbal measures of visual attention span demonstrated significant correlations. However, only verbal visual attention was significantly correlated with reading fluency. The relationship between visual attention span and logographic languages among adults was also examined, thereby addressing the limitations of previous studies and furthering the research in this area.

## Data Availability Statement

The datasets for this manuscript are not publicly available because of the approved ethical condition of the study. Requests to access the datasets should be directed to KC, kevinsc@hku.hk.

## Ethics Statement

The studies involving human participants were reviewed and approved by the Human Research Ethics Committee of the University of Hong Kong. The patients/participants provided their written informed consent to participate in this study.

## Author Contributions

KC organized the database, performed the statistical analysis, and wrote the first draft of manuscript. Both authors contributed to the design of the study, the revision of the manuscript, and read and approved the submitted version.

## Conflict of Interest

The authors declare that the research was conducted in the absence of any commercial or financial relationships that could be construed as a potential conflict of interest.
